# Clinical prediction nomogram for steroid-induced ocular hypertension risk in patients with intravitreal dexamethasone implant

**DOI:** 10.1016/j.heliyon.2024.e34635

**Published:** 2024-07-15

**Authors:** Won Jeong Cho, Hye Jung Shin, Min Kim, Hyoung Won Bae, Chan Yun Kim, Wungrak Choi

**Affiliations:** a Institute of Vision Research, Department of Ophthalmology, Yonsei University College of Medicine, Seoul, South Korea; b Biostatistics Collaboration Unit, Department of Biomedical Systems Informatics, Yonsei University College of Medicine, Seoul, South Korea

**Keywords:** Steroid-induced ocular hypertension, Intraocular pressure, Trabecular meshwork height, Anterior segment optical coherence tomography, Intravitreal dexamethasone injection

## Abstract

**Background:**

Recognizing the risk factors and understanding the mechanisms underlying steroid-induced ocular hypertension (SIOH) are vital to prevent potent vision loss and ensure the safety and effectiveness of dexamethasone (DEX) injections. The study aimed to develop a novel nomogram for predicting the risk of SIOH and determining safety zones for steroid injections.

**Methods:**

This single-center, retrospective, case-control study included a total of 154 eyes with available measured axial length that had undergone AS-OCT and DEX implantation at the Yonsei University Health System. The eyes were categorized into the SIOH (n = 39) and post-steroid normal IOP (n = 115) groups. We measured intraocular pressure (IOP) for all eyes prior to DEX implantation, at 1 week post-implantation, and at 1, 2, 3, 6, and 12 months thereafter. We used AS-OCT to analyze the trabecular meshwork (TM) height and ocular parameters.

**Results:**

The predictive nomogram, including TM height, yielded an AUC of 0.807 (95 % confidence interval [CI], 0.737–0.877) and demonstrated significantly higher predictive accuracy than that of previous nomograms, which did not consider TM height and had an AUC of 0.644 (95 % CI, 0.543–0.745) (*p* = 0.031). The calibration plot demonstrated a strong predictive accuracy for a predicted value of approximately 0.4. We established cutoff values to ensure different levels of sensitivity and specificity within the safety zone following DEX implantation.

**Conclusion:**

Our improved nomogram incorporating TM height as a newly identified risk factor, established a safety threshold for intravitreal DEX implantation, helping identify safe individuals from those who require caution.

## 1 Introduction

Steroid-induced ocular hypertension (SIOH) is characterized by an elevated intraocular pressure (IOP) ≥25 mmHg or an increase ≥10 mmHg from the baseline value within the affected eye. SIOH occurs as a result of prolonged use or misuse of systemic or topical corticosteroid medications [[1], [2], [3], [4], [5], [6]]. In 1950, McLean reported elevated IOP induced by the systemic administration of adrenocorticotrophic hormone (ACTH). Subsequently, Francosis reported the first case of increased IOP caused by the topical application of cortisone in 1954 [7,8]. A previous study revealed that SIOH was observed in 28.5 % of eyes receiving steroid injections. However, SIOH may go undetected and untreated for a long duration after the elevated IOP reaches a significant magnitude in 9.5 % of individuals, leading to the development of glaucomatous optic neuropathy, known as steroid-induced glaucoma [[9], [10], [11], [12]]. Consequently, this specific type of secondary ocular hypertension has garnered attention both as a standalone condition and potential source of insights into the causes of certain forms of open-angle glaucoma (OAG) [2,3,13].

Nevertheless, benefits of anti-inflammatory effects of corticosteroid therapy, with careful and judicious management, may outweigh the risks of SIOH. Intravitreal dexamethasone (DEX) injection is an exemplary treatment option for various ocular conditions, particularly those involving inflammation, macular edema, and certain retinal disorders [[14], [15], [16]]. Intravitreal DEX injections enable the administration of potent corticosteroids such that high intravitreal and retinal concentrations can be achieved immediately, in addition to a sustained release over a 6-month period [2,10,11].

Recognizing the risk factors and understanding the mechanisms underlying SIOH are vital to prevent potent vision loss and ensure the safety and effectiveness of DEX injections. Multiple predisposing risk factors have been identified. Trabecular meshwork (TM) height is one such newly discovered risk factor. It is defined as the distance between the scleral spur and Schwalbe's line. The scleral spur is a landmark structure formed from a projection of the sclera, while Schwalbe's line represents the end of the corneal endothelium and the beginning of the trabecular meshwork. This measurement can be obtained noninvasively using anterior segment optical coherence tomography (AS-OCT) [17,18]. TM plays a central role in regulating the drainage of aqueous humor. Thus, patients with a shorter TM height should be treated with caution as an increase in cell density in this structure due to the administration of steroids may result in increased TM shrinkage [[19], [20], [21]]. However, only few studies have focused on the prediction of SIOH. Therefore, this study aimed to develop a novel nomogram to predict the risk of SIOH and identify the safety zones for the administration of steroid injections to improve the accuracy of SIOH prediction.

## 2 Methods

### 2.1 Patient enrollment

This retrospective case-control study enrolled 243 patients (251 eyes) who underwent intravitreal DEX (OZURDEX; Allergan, Inc., Irvine, CA, USA) implantation of 0.7 mg and AS-OCT between March 2013 and May 2022 at the Yonsei University Health System (Seoul, South Korea). A single DEX implant was used for the treatment of several ophthalmic diseases, including diabetic macular edema (DME), retinal vein occlusion (RVO), and uveitis. Patients who met the following inclusion criteria were included in this study: available axial length (AXL) measurement; IOP followed up for up to 12 months post-injection; and complete medical records, including data regarding age, sex, laterality of eyes, primary diagnosis necessitating DEX implantation, underlying medical conditions, and ocular exam results available for review. Eyes with open angles on AS-OCT and glaucomatous eyes, such as primary open-angle glaucoma (POAG), glaucoma suspect, and secondary glaucoma, were included in this study. Glaucomatous eyes were characterized by the presence of changes in the optic nerve head, such as focal nerve fiber layer defects, optic cup size alterations, neural rim changes, optic nerve hemorrhages, increase in the cup-disc (C/D) ratio, or differences between eyes in various glaucoma types [22].

We excluded 36 eyes with poor-quality AS-OCT images that prevented the measurement of the ocular parameters. In addition, we also excluded 11 eyes with a baseline IOP ≥23 mmHg, 15 eyes with primary angle closure glaucoma (PACG) or primary angle closure (PAC), 30 eyes with appositional angle closure (i.e., eyes with a closed angle on the nasal or temporal side of an eye owing to irido-trabecular contact [23,24]), 2 eyes with a history of penetrating trauma, and 3 eyes with systemic complications from systemic corticosteroid therapy. Thus, 154 eligible eyes were included in the final analysis.

### 2.2 Study design

The 154 eligible eyes were divided into a post-DEX normal IOP (Group A, 115 eyes) and SIOH groups (Group B, 39 eyes) (Table 1). Data regarding age, sex, laterality, diagnosis necessitating DEX implantation, underlying systemic disease, glaucoma history, and ocular exam results were extracted from the medical records (Table 1). Based on the findings of previous studies, we defined SIOH as an IOP ≥25 mm Hg following intravitreal DEX injection(15/39 eyes with SIOH) or IOP increase ≥10 mm Hg following the intravitreal DEX injection compared with the baseline IOP (24/39 eyes with SIOH) [[1], [2], [3], [4], [5], [6]]. The patients underwent eye evaluations before and after the intravitreal injection DEX.

**Table 1:** Demographic characteristics of the study population.

Baseline Characteristic	All Eyes (N = 154 eyes)	Patients without SIOH^a^Group A (n = 115 eyes)	Patients with SIOH Group B (n = 39 eyes)	p-value
Age (years)	60 (52,67)	61 (52,69)	58 (50,63)	0.120
Sex (M/F) (patients)	76 (49.35 %)/78 (50.65 %)	53 (46.09 %)/62 (53.91 %)	23 (58.97 %)/16 (41.03 %)	0.164†
Laterality (R/L) (eyes)	90 (58.44 %)/64 (41.56 %)	70 (60.87 %)/45 (39.13 %)	20 (51.28 %)/19 (48.72 %)	0.294†
Diagnosis for DEX implants (eyes)				
DME	47 (30.52 %)	42 (36.52 %)	5 (12.82 %)	0.045^c^
RVO	48 (31.17 %)	34 (29.57 %)	14 (35.90 %)	
Uveitis	26 (16.88 %)	17 (14.78 %)	9 (23.08 %)	
Others	33 (21.43 %)	22 (19.13 %)	11 (28.21 %)	
AXL (mm)	23.42 (22.8, 24.51)	23.21 (22.74, 24.35)	24.08 (23.26, 24.99)	0.002^c^
SE (D)	−0.25 (−1.75,0.5)	−0.25 (−1.75, 0.75)	−0.75 (−1.75,0.25)	0.248
CCT (μm)	560 (532, 600)	558 (531, 601)	564 (533, 600)	0.571
Baseline IOP (mmHg)	13 (11,15)	13 (11,15)	14 (12,17)	0.025*
Systemic disease				
Hypertension	56 (36.36 %)	43 (37.39 %)	13 (33.33 %)	0.649†
Thyroid disease	5 (3.25 %)	5 (4.35 %)	0 (0.0 %)	0.331§
Kidney disease	6 (3.90 %)	4 (3.48 %)	2 (5.13 %)	0.643§
Cancer	6 (3.90 %)	5 (4.35 %)	1 (2.56 %)	1.000§
Diabetes mellitus (type2)	71 (46.10 %)	59 (51.30 %)	12 (30.77 %)	0.026†^c^
Cardiovascular problem	15 (9.74 %)	12 (10.43 %)	3 (7.69 %)	0.762§
Cerebrovascular accident	7 (4.55 %)	5 (4.35 %)	2 (5.13 %)	1.000§
Connective tissue disease	7 (4.55 %)	5 (4.35 %)	2 (5.13 %)	1.000§
Glaucoma				
Primary open angle glaucoma	3 (1.95 %)	2 (1.74 %)	1 (2.56 %)	1.000§
Glaucoma suspect^b^	9 (5.84 %)	7 (6.09 %)	2 (5.13 %)	1.000§
Secondary glaucoma	10 (6.49 %)	6 (5.22 %)	4 (10.26 %)	0.275§

Patients who received 0.7 mg dexamethasone (DEX) implants were recruited. Continuous variables did not meet the normality assumption; therefore, they were analyzed using non-parametric methods (median (Q1,Q3), and Mann–Whitney *U* test p-value. Categorical variables are presented using descriptive statistics as numbers (%) using †χ2 test; §Fisher's exact testAXL, axial length; CCT, central corneal thickness; DEX, dexamethasone; IOP, intraocular pressure; SE, spherical equivalent; SIOH, steroid-induced ocular hypertension; DME, diabetic macular edema; RVO, retinal vein occlusion.

a The SIOH group was divided as follows: post-injection IOP ≥25 mmHg or IOP elevation ≥10 mmHg over the baseline measurement.

b Glaucoma suspect was defined as changes in the optic nerve head, including generalized or focal increases in the optic cup size and increases >0.6 in the cup-disc ratio; narrowing or notching of the neural rim; optic nerve hemorrhaging; and a cup-disc ratio asymmetry >0.2 between the two eyes.

c statistical significance.

### 2.3 Clinical measurements

We measured AXL, IOP, and TM height similarly with the procedures in our previous studies [17,25]. AXL measurements of the included eyes were obtained using IOL Master (Carl Zeiss Meditec AG, Jena, Germany). We used a Goldmann applanation tonometer (GAT) to measure the IOP before and after DEX implantation; the baseline IOP was measured before administering steroid injection [26,27]. The IOP was assessed at seven time points: at baseline; 1 week post-injection; and 1, 2, 3, 6, and 12 months post-injection. The final IOP was calculated as the average of three consecutive measurements in a single session. Additional measurements were obtained for 19 eyes as the difference between the initial two measurements was >1 mmHg. We used CASIA SS-1000 AS-OCT (Tomey Corporation, Nagoya, Japan) and AS-OCT images acquired closest in time after treatment to measure TM height and various ocular parameters. Using the standard protocols from prior studies, we defined TM height as the distance between the scleral spur and Schwalbe's line [17,28,29]. Automated analyses of the ocular parameters, including anterior chamber depth (ACD), anterior chamber width (ACW), lens vault (LV), angle opening distance (AOD), angle recess area (ARA), trabecular iris space area (TISA), and trabecular iris angle (TIA) measured at 500 μm from the scleral spur, were performed by identifying the scleral spur [30]. We used the average of the nasal and temporal side of an eye for all variables. The central corneal thickness (CCT) was evaluated by manually measuring the corneal width on cross-sectional AS-OCT images.

### 2.4 Statistical analyses

Data analyses, visualization, and logistic regressions for the nomogram were performed using SPSS V22.0 software (SPSS, Chicago, IL, USA), SAS version 9.4 (SAS Institute Inc., Cary, NC, USA), and R package (version 4.3.0, packages; survival; The R Project for Statistical Computing, Vienna, Austria). Continuous variables are presented as mean ± standard deviation or median (Q1, Q3), whereas categorical variables are presented as the number of patients (percentage). For continuous variables, we employed parametric (p-value from Student's t-test) or non-parametric methods (p-value from Mann–Whitney *U* test) to analyze the between-group differences based on normality test result. For categorical variables, we used the chi-squared or Fisher's exact test, with descriptive statistics presented as numbers (%). Fisher's exact test was used if the proportion of cells with an expected frequency of <5 was >20 % of the total cells.

Logistic regression analysis was used to calculate the odds ratios (ORs) for SIOH. The variables were categorized into three sets of potential risk factors for SIOH: ocular parameters (TM height, ACD, ACW, AOD, ARA, TISA, TIA, and LV), baseline characteristics (age, baseline IOP, sex, laterality, AXL, spherical equivalent (SE), CCT, and diagnosis necessitating DEX implant), and systemic disease (hypertension, kidney disease, cancer, type 2 diabetes mellitus [DM], cardiovascular problems, cerebrovascular accidents, and connective tissue disease). The categorized variables were used for logistic regression. Significant variables from the univariable analysis, in addition to those previously identified as significant in previous studies, were used in a multivariable model to devise a predictive nomogram. We categorized continuous variables in the multivariable model analysis according to the convenience of nomogram users. We used Youden's index, assigning equal weight to the sensitivity and specificity to maximize the J index (J = max(sensitivity + specificity −1)) to determine the optimal cutoff points for baseline IOP, AXL, and TM height. Age was categorized into <40 years ≥ 80 years, and intervals of 10 years in between. Model performance was assessed using the area under the receiver operating characteristic (ROC) curves and calibration plots. We provided the optimal cutoff point for the nomogram score and points that attained a sensitivity and specificity of at least 80 %, 85 %, 90 %, and 95 %. We applied the DeLong method for the area under the curve (AUC) using the current data to compare the predictive performance of the proposed nomogram with that of the previous research model.

## 3 Results

Table 1 summarizes the baseline characteristics of the patients who received intravitreal DEX implants. The baseline IOP, AXL, presence of type 2 DM, primary diagnosis necessitating DEX implantation, and TM height differed significantly between the groups (p < 0.05; Table 1, Table 2). A high baseline IOP, long AXL, absence of type 2 DM, primary diagnosis necessitating DEX implantation, and short TM height showed a significant association with SIOH development in the univariable logistic regression analysis (p < 0.05; Table 3). TM height and baseline IOP were significant variables in the multivariable analysis (p < 0.05; Table 3).

**Table 2:** Comparison between ocular parameters of patients enrolled in Groups A and B.

Ocular parameters	All Eyes (N = 154 eyes)	Patients without SIOH Group A (n = 115 eyes)	Patients with SIOH Group B (n = 39eyes)	p-value
TM height (μm)	772.5 (717.5, 820)	787.5 (733.75, 830)	733 (701.25, 776)	<0.001^a^
ACD (mm)	3.3 (3.1, 3.52)	3.33 (3.12, 3.52)	3.26 (3.07, 3.51)	0.540
ACW (mm)	11.44 ± 0.43	11.45 ± 0.41	11.41 ± 0.47	0.576^b^
AOD (μm)	584 (450, 715)	581 (468, 710)	589 (410, 727.5)	0.961
ARA (μm)	230 (175, 294)	236 (171, 298)	218.5 (175, 283.5)	0.682
TISA (μm)	218 (166, 271.5)	218.5 (164.5, 271.5)	214.5 (166, 272)	0.931
TIA (μm)	52.85 ± 13.60	52.63 ± 13.97	53.49 ± 12.66	0.737^b^
LV (mm)	−0.28 (−0.44, −0.02)	−0.31 (−0.45, −0.03)	−0.23 (−0.36, −0.02)	0.583

All values are averaged with the measurements performed at both temporal and nasal sides of eyes; ACD, anterior chamber depth; ACW, anterior chamber width; AOD, angle opening distance; ARA, angle recess area; LV, lens vault; SIOH, steroid-induced ocular hypertension; TIA, trabecular iris angle; TISA, trabecular iris space area; TM, trabecular meshwork.

a p < 0.05 from *t*-test.

b Variables analyzed using parametric methods (mean, standard deviation, and Student's t-test p-value).

**Table 3:** Univariable and multivariable logistic regression of variables that may cause SIOH.

Variables	N	Univariable analysis Multivariable analysis			
		OR (95 % CI)	p-value	OR (95 % CI)	p-value
Ocular parameters					
TM height (μm)^a^	154	0.990 (0.984, 0.996)	0.001^a^	0.992 (0.986, 0.999)	0.024^a^
ACD (mm)	154	0.783 (0.322, 1.908)	0.591		
ACW (mm)	154	0.782 (0.331, 1.848)	0.575		
AOD (μm)	154	1.000 (0.999, 1.001)	0.973		
ARA (μm)	154	0.999 (0.996, 1.002)	0.491		
TISA (μm)	154	1.001 (0.997, 1.005)	0.623		
TIA (μm)	154	1.005 (0.978, 1.032)	0.735		
LV (mm)	154	1.210 (0.445, 3.287)	0.709		
Baseline characteristics					
Age	154	0.986 (0.959, 1.015)	0.3499		
Baseline IOP (mmHg)^a^	154	1.116 (1.035, 1.205)	0.004^a^	1.108 (1.016, 1.209)	0.021^a^
Sex (Female)	154 (78)	0.595 (0.285, 1.241)	0.166		
Laterality (Left)	154 (64)	1.478 (0.711, 3.070)	0.295		
AXL (mm)^a^	154	1.427 (1.100, 1.851)	0.007^a^	1.226 (0.936, 1.607)	0.139
SE (D)	154	0.928 (0.814, 1.057)	0.260		
CCT (μm)	154	1.003 (0.997, 1.010)	0.325		
Diagnosis for DEX implant (eyes)					
DME	154	ref		ref	
RVO	154	3.458 (1.132, 10.563)	0.029^a^	2.156 (0.505, 9.201)	0.299
Uveitis	154	4.446 (1.300, 15.207)	0.017^a^	1.978 (0.362, 10.823)	0.432
Others	154	4.199 (1.295, 13.613)	0.017^a^	1.761 (0.383, 8.103)	0.468
Systemic disease					
HTN	154	0.837 (0.389, 1.800)	0.649		
Kidney disease	154	1.500 (0.264, 8.527)	0.647		
Cancer	154	0.579 (0.066, 5.114)	0.623		
DM (type2)^a^	154	0.422 (0.195, 0.913)	0.028^a^	0.552 (0.172, 1.776)	0.319
Cardiovascular problem	154	0.715 (0.191, 2.680)	0.619		
CVA	154	1.189 (0.221, 6.391)	0.840		
Connective tissue disease	154	1.189 (0.221, 6.391)	0.840		

SIOH; steroid-induced ocular hypertension; OR, odds ratio; CI, confidence interval; TM, trabecular meshwork; ACD, anterior chamber depth; ACW, anterior chamber width; AOD, angle opening distance; ARA, angle recess area; TISA, trabecular iris space area; TIA, trabecular iris angle. LV, lens vault; IOP, intraocular pressure; AXL, axial length; SE, spherical equivalent; CCT, central corneal thickness; HTN, hypertension; DM, diabetes mellitus; CVA, cerebrovascular accident.

a Statistical significance.

### 3.1 Multivariable model results

**Fig. 1:**
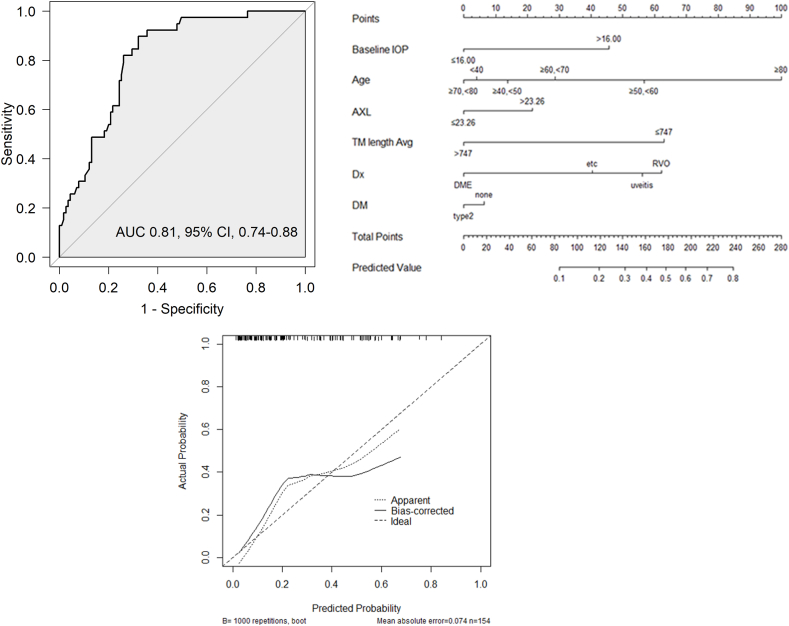
**Nomogram of the multivariable model** **A. Receiver operating characteristic curve plot of the multivariable model** (A) The area under the receiver operating characteristic curve of multivariable model 1 is 0.807. **B. Nomogram of the multivariable model** (B) The prediction probability was calculated using the following formula: prediction probability = 1/(1+exp(-(-3.9220 + 1.0675 × baseline_IOP (>16) + 0.2270 × age (≥40, <50) + 1.2303 × age (≥50,<60) + 0.5780 × age (≥60, <70) - 0.0941 × age (≥70, <80) + 2.2433 age (≥80) + 0.5017 AXL (>23.26) + 1.4727 × TM height (≤747) + 1.4531 × Dx (=RVO) + 1.3126 Dx (=uveitis) + 0.9435 × Dx(=etc.) - 0.1480 × DM (=type2))) **C. Calibration plot of the multivariable model** (C) Calibration plot of the multivariable model visually demonstrating the accuracy of the predicted probabilities obtained from the prediction model with actual probabilities. The dashed line (apparent) represents the original dataset results, whereas the solid line (bias-corrected) depicts the results from internal validation using bootstrap samples. The calibration line is above the diagonal dashed line up to a probability of approximately 0.4, indicating underestimation. The calibration line is below the diagonal dashed line beyond the probability of 0.4, suggesting overestimation.

We devised a multivariable model for predicting the probability of SIOH by including all significant variables in the univariable regression, along with a variable (age) known to be significant from prior studies (Table 4). Elevated baseline IOP (>16 mmHg) and shorter TM height (≤747 μm) showed significant associations with SIOH. The AUC of the model was 0.8071 (95 % confidence interval [CI] 0.737–0.877) (Fig. 1A). The p-value of the Hosmer–Lemeshow goodness-of-fit test was 0.403, indicating that the model provided a good fit to the data (p > 0.05; Table 4). In contrast to the previous nomogram [25] (AUC of 0.644, 95 % CI 0.543–0.745), the new nomogram exhibited a significant difference in predictive performance, as indicated by a p-value of 0.003 (Table 5).

**Table 4:** Multivariable model of variables that may cause SIOH.

		Odds Ratio (95 % CI)			p-value
		OR	lower	upper	
Baseline IOP b	≤16.00	ref			–
	>16.00	2.908	1.004	8.428	0.049*
Age	<40	ref			–
	≥40, <50	1.255	0.130	12.120	0.844
	≥50, <60	3.422	0.437	26.798	0.241
	≥60, <70	1.782	0.215	14.747	0.592
	≥70, <80	0.910	0.079	10.538	0.940
	≥80	9.424	0.505	175.756	0.133
AXL b	≤23.26	ref			–
	>23.26	1.652	0.636	4.290	0.303
TM height b	>747	ref			–
	≤747	4.361	1.697	11.208	0.002^a^
Diagnosis for DEX implant	1. DME	ref			–
	2. RVO	4.276	0.920	19.883	0.064
	3. Uveitis	3.716	0.620	22.269	0.151
	4. Others	2.569	0.566	11.666	0.222
DM	None	ref			–
	Type2	0.862	0.282	2.642	0.796
AUC (95 % CI)		0.807 (0.737, 0.877)			
Hosmer–Lemeshow Goodness-of-Fit Test p-value		0.403			

SIOH, steroid-induced ocular hypertension; OR, odds ratio; CI, confidence interval; IOP, intraocular pressure; TM, trabecular meshwork; DEX, dexamethasone; DM, diabetes mellitus; AUC, area under the curve.

a Statistical significance.

b Variables that used optimal cutoff points for binary variable analysis.

**Table 5:** Comparison with previous nomogram using the Delong method.

	Nomogram 2023	Nomogram 2020^a^	p-value
AUC (95 % CI)	0.807 (0.737, 0.877)	0.644 (0.54^a^, 0.745)	0.031^b^

a Nomogram from a previous study *(Choi W* et al. *Br J Op*^*a*^*thalmol 2022;106:1150–1156)* AUC, area under the curve; CI, confidence interval.

b Statistical significance.

### 3.2 Nomogram of multivariable model

The prediction probability formula for the multivariable model is as follows:

Prediction probability = 1/(1+exp(-(-3.9220 + 1.0675 × baseline IOP (>16) + 0.2270 × age (≥40,<50) + 1.2303 × age (≥50, <60) + 0.5780 × age (≥60, <70) - 0.0941 × age (≥70, <80) + 2.2433 × age (≥80) + 0.5017 × AXL (>23.26) + 1.4727 × TM height (≦747) + 1.4531 × Dx (=RVO) + 1.3126 × Dx (=uveitis) + 0.9435 × Dx (=etc.) - 0.1480 × DM (=type2))) (Table 6).

**Table 6:** Nomogram points and predictive value of the multivariable model.

Baseline IOP^a^	Points	Age	Points	AXL^a^	Points	TM height†	Points	Dx	Po^a^nts	DM	Points	Total points	Predicted val^a^e
≤16.00	0	<40	4	≤23.26	0	>747	0	DME	0	none	6	84	0.1
>16.00	46	≥40, <50	14	>23.26	21	≤747	63	RVO	62	type2	0	119	0.2
		≥50, <60	57					uveitis	56			142	0.3
		≥60, <70	29					others	40			161^b^	0.4^b^
		≥70, <80	0									178	0.5
		≥80	100									1^b^5	0.^b^
												214	0.7
												237	0.8

AXL, axial length; TM, trabecular meshwork; Dx, diagnosis for dexamethasone implantation; DME, diabetic macular edema; RVO, retinal vein occlusion; DM, diabetes mellitus.

a Variables that used optimal cut-off points for binary variable analysis.

b indicates accurate prediction up to a probability of approximately 0.4 (total points 161).IOP, intraocular pressure.

A calibration plot generated to assess the alignment of the predicted probabilities with the actual probabilities revealed that the prediction was accurate up to a probability of approximately 0.4 (total points 161). The calibration line positioned above the diagonal dashed line indicated a tendency toward underestimation, whereas that positioned beyond 0.4, which was below the diagonal dashed line, indicated a tendency toward overestimation (Fig. 1C and Table 5).

### 3.3 Cutoff values of prediction nomograms

The optimal cutoff point was 119, yielding a sensitivity of 87.18 % and specificity of 68.70 %, with the highest Youden index of 0.56. Various threshold values for clinical application were offered in a range of sensitivities and specificities (Table 7).

**Table 7:** Cutoff points of the nomogram scores.

Cutoff point				
Nomogram score	Sensitivity		Specificity	Youden Index
≤71, >71	–	100.00 %	37.39 %	0.37
≤76, >76	–	97.44 %	38.26 %	0.36
≤98, >98	–	92.31 %	53.91 %	0.46
≤119, >119^a^	Optimal	87.18 %	68.70 %	0.56
≤124, >124	–	82.05 %	70.43 %	0.53
≤159, >159	–	53.85 %	80.87 %	0.35
≤166, >166	–	43.59 %	85.22 %	0.29
≤181, >181		33.33 %	91.30 %	0.25
≤187, >187		23.08 %	96.52 %	0.20
≤203, >203		12.82 %	99.13 %	0.12

a The “optimal” cutoff value was defined by the highest Youden index value (sensitivity + specificity-1) from logistic regression.

## 4 Discussion

This study aimed to develop a novel nomogram to predict the risk of SIOH and identify the safety zones for the administration of steroid injections to improve the accuracy of SIOH prediction. We improved the accuracy of the nomogram in predicting the risk of SIOH in eyes that underwent intravitreal DEX implantation to enable better risk assessment. The novelty of our study lies in that the proposed nomogram incorporated AS-OCT and included TM height, a newly identified risk factor that can be measured noninvasively, in addition to the existing risk factors, such as high myopia, a history of previous SIOH, young age, POAG, a first-degree relative with POAG, presence of type 1 DM, connective tissue disease, and penetrating keratoplasty [12,31,32]. Using AS-OCT for evaluating the ocular anatomy in SIOH patients and employing the nomogram for predicting SIOH before the intravitreal DEX injections can help physicians prevent unwanted IOP elevations, manage inflammation, and assist patients requiring steroid treatment. The implications of our study are clinically significant, given the frequent occurrence of SIOH and its potential to cause severe visual impairment [13].

Our univariable analysis results suggested that steroid treatment should be initiated with caution in patients with elevated baseline IOP; extended AXL; no history of type 2 DM; primary diagnosis for DEX implant (DME, RVO, uveitis, and other conditions that necessitate DEX implantation); and shorter TM height. Our multivariable analysis revealed that elevated baseline IOP and shorter TM height had a significant effect on SIOH development, which is consistent with the findings of a previous study [17].

The age range (6–88 years) for intravitreal DEX implantation in our study was broad, and this enabled a detailed comparison of SIOH risks by age. Although age showed no significant results in this study owing to the smaller sample size (154 eyes) compared with that of a previous study (908 eyes), we included young age as a variable since it was a known a risk factor for SIOH in our previous studies [33,34]. Our findings also indicated that baseline IOP could be considered another contributing factor to SIOH. These results align with the understanding that POAG is an established individual risk factor associated with post-steroidal IOP elevation, thereby reinforcing our hypothesis that individuals with higher baseline IOP may be more susceptible to developing POAG owing to increased susceptibility to SIOH [35,36].

Notably, type 2 DM showed protective effect in the univariable analysis, with an OR of 0.422 (0.195, 0.913, p = 0.028), whereas type 1 DM was a risk factor for SIOH. Evidence suggests that metformin, which is commonly used for type 2 DM, may mediate the protective effects against POAG. The administration of metformin as an initial therapy for type 2 DM is recommended owing to its neuroprotective properties [37]. A retrospective study of 150,016 patients with diabetes revealed a dose-dependent reduction in POAG risk with metformin use, even when considering glycemic control. Each 1 g increase in metformin dose was associated with a reduction of 0.16 % in the risk for POAG. Furthermore, receiving 2 g of metformin daily for 2 years potentially reduces POAG risk by 20.8 % [[37], [38], [39], [40]]. Akkaya et al. also reported a protective effect of DM against optic nerve damage in patients with POAG, indicated by the significantly higher optic nerve head rim area and rim volume in POAG patients with DM than those of patients without DM [41]. Notably, most patients with diabetes in our study had type 2 DM (71 eyes), which might partly account for this outcome (Table 3). However, the relationship between glaucoma and diabetes is a complex interplay of various factors, including genetics, vascular changes, and IOP [38]. Thus, these findings must be interpreted with caution, as the relationship between DM and the prevalence of glaucoma remains inconsistent. More objective and precise data regarding the diagnoses of the participants are required for future studies.

The insufficient number of eyes with AXL measurements was a limitation of our previous studies (470 of 908 eyes [25] and 74 of 102 eyes [17] with AXL measurements). Therefore, we included all 154 eyes with AXL measurements in this study, and a long AXL was significant associated with SIOH in both univariable and multivariable analyses, with ORs of 1.427 (1.100, 1.851, p = 0.007) and 1.226 (0.936, 1.607, p = 0.139), respectively. AXL was also dichotomized based on the optimal cutoff point determined using the Youden Index (23.26 mm) and included in the multivariable model for user convenience. Our findings are consistent with those of a previous study that identified an association between high myopia and SIOH [42].

Along with filling the gap regarding AXL, the addition of TM height to the nomogram significantly improved its predictive accuracy. TM, a novel risk factor that can be readily measured in clinical practice using AS-OCT, was identified as the core anatomical structure regulating the aqueous outflow pathway in our previous study [17,20,43]. A shorter TM height was a significant variable in univariable (OR 0.990; 0.984, 0.996; p = 0.001) and multivariable analyses (OR 0.992; 0.986, 0.999; p = 0.024). Patients with a TM height ≤747 μm, the optimal cutoff point, had an OR of 4.361 (1.697, 11.208; p = 0.002) in the multivariable model.

We assessed the improvement in the predictive performance of the nomogram by comparing the AUCs of the previous and current versions using the current dataset [25]. The new nomogram demonstrated a significant improvement in predictive performance, with a p-value of 0.0031 (Table 5). We also evaluated the alignment of predicted probabilities with actual probabilities using a calibration plot and found a strong predictive performance, particularly for values of up to 0.4. This suggests that the total nomogram scores below 161 provided accurate predictions (Fig. 1B and Table 6).

We also aimed to predict the likelihood of SIOH and establish a suitable safety zone for DEX injections. We calculated the optimal cutoff value as 119 points, yielding a relatively high sensitivity of 87.18 %, effectively identifying true positive cases when SIOH is present and a moderate specificity of 68.70 %, indicating that it correctly identifies true negative cases but also leads to a notable rate of false positives (Table 7). The highest Youden index of 0.56 indicated a reasonable balance between sensitivity and specificity, optimizing our chosen cutoff value for overall predictive performance. When compared to the latest existing predictive model for SIOH [25], which had sensitivity and specificity values of 68.42 % and 59.10 %, respectively, our test demonstrated improved performance. Furthermore, we provided threshold values to meet the minimum level of sensitivity and specificity (80 %, 85 %, 90 %, and 95 %) to facilitate clinical application. For instance, when the nomogram cutoff value is set at 98 for diagnosing positive and negative results, the probability of obtaining a positive result with SIOH is 92 %. Similarly, on setting the cut-off value for diagnosing SIOH at 181, the specificity at that point was 91.30 %. This indicates that patients without SIOH had a 91.3 % probability of receiving a negative diagnostic result. Clinicians may feel more at ease in using steroid treatment for various vitreoretinal conditions in such cases, and monitoring for potential SIOH during clinic visits may be more manageable.

As steroid-induced glaucoma may result in vision threats or even blindness, recognizing the risk factors and understanding the mechanisms underlying SIOH are vital to preventing significant vision loss and ensuring the safety and effectiveness of DEX injections. Establishing a nomogram to predict the risk of increased IOP in these patients allows for individualized treatment planning. Specifically, the nomogram developed in this study enables clinicians to predict the risk of SIOH with greater accuracy, allowing them to identify patients who can safely receive intravitreal dexamethasone (DEX) injections and those who require closer monitoring or alternative treatments.

We do not know the exact mechanism of why TM height plays a significant role in SIOH. However, there may be several possibilities that should be explored in the near future. Selective laser trabeculoplasty (SLT) works selectively on the trabecular meshwork, inducing remodeling and promoting aqueous outflow, thereby lowering intraocular pressure [[44], [45], [46], [47]]. This mechanism of SLT may help explain why the accuracy of our new nomogram increased with the inclusion of TM height. A shorter TM length indicates a smaller trabecular meshwork, which must overwork to maintain adequate outflow and becomes more susceptible to stress. In this regard, we can consider SLT as a preventive treatment option for high-risk patients identified by the nomogram, particularly those with a small TM height. However, there is a lack of research on whether the prophylactic effects of SLT are significant concerning steroid-induced changes in the trabecular meshwork, indicating the need for further studies in the future.

The limitations of our study included its retrospective design, the unequal and insufficient number of patients included in the groups, and the inability to perform a direct comparison of our results with those of prospective randomized controlled trials. We categorized continuous variables for user convenience; however, the nomogram points did not follow a consistent pattern as the number of categories increased and appeared disordered for some variables. This finding may be attributed to the distribution of sample sizes and event counts in the data. Thus, we aim to collect additional data for future research to reanalyze and address these inconsistencies. Despite these limitations, a significant strength of this study lies in the development of an improved nomogram formula that incorporates a recently identified risk factor, TM, which can be measured noninvasively. The inclusion of TM has enhanced the robustness of our conclusions.

In conclusion, our nomogram established a safety threshold for intravitreal DEX implantation. Patients within this safety zone can receive DEX with increased assurance, whereas those with high nomogram scores warrant cautious consideration when prescribing DEX. We recommend conducting a large-scale prospective randomized study in the future to validate our findings.

### Funding/support

This work was supported by the Basic Science Research Program through the 10.13039/501100003725National Research Foundation of Korea (NRF- 2022R1I1A1A01071919), the Research Grant from Gangnam Severance Hospital (D-2023-0012), and by a new faculty research seed money grant of 10.13039/501100008005Yonsei University College of Medicine for 2023 (2023-32-0059). The funding organization had no role in the design or conduct of this research.

### Financial disclosures

No financial disclosures. All authors attest that they meet the current ICMJE criteria for authorship.

## Data availability

The datasets used and/or analyzed in the current study are available from the corresponding author upon reasonable request.

### Ethics statement

The Institutional Review Board (IRB) of the Gangnam Severance Hospital approved this study and waived the requirement for obtaining informed consent from the patients as a review of existing patient records was performed in this study (IRB number: 2023-0477-001). The study protocol adhered to the tenets of the Declaration of Helsinki and complied with the Health Insurance Portability and Accountability Act.

## CRediT authorship contribution statement

**Won Jeong Cho:** Writing – review & editing, Writing – original draft, Formal analysis, Data curation, Conceptualization. **Hye Jung Shin:** Formal analysis, Data curation. **Min Kim:** Investigation, Conceptualization. **Hyoung Won Bae:** Methodology, Investigation. **Chan Yun Kim:** Validation, Supervision, Conceptualization. **Wungrak Choi:** Writing – review & editing, Writing – original draft, Resources, Investigation, Conceptualization.

## Declaration of competing interest

The authors declare that they have no known competing financial interests or personal relationships that could have appeared to influence the work reported in this paper.
